# Genome-wide identification, phylogeny, evolutionary expansion and expression analyses of *bZIP* transcription factor family in tartaty buckwheat

**DOI:** 10.1186/s12864-019-5882-z

**Published:** 2019-06-11

**Authors:** Moyang Liu, Yongdi Wen, Wenjun Sun, Zhaotang Ma, Li Huang, Qi Wu, Zizhong Tang, Tongliang Bu, Chenglei Li, Hui Chen

**Affiliations:** 1 Sichuan Agricultural University, College of Life Science, Ya’an, China; 2 Shanghai Jiao Tong University, School of Agriculture and Biolog, Shanghai, China

**Keywords:** *FtbZIP*, Tartary buckwheat, Development, Expression patter, Fruit, ABA

## Abstract

**Background:**

In reported plants, the *bZIP* family is one of the largest transcription factor families. *bZIP* genes play roles in the light signal, seed maturation, flower development, cell elongation, seed accumulation protein, abiotic and biological stress and other biological processes. While, no detailed identification and genome-wide analysis of *bZIP* family genes in *Fagopyum talaricum* (tartary buckwheat) has previously been published. The recently reported genome sequence of tartary buckwheat provides theoretical basis for us to study and discuss the characteristics and expression of *bZIP* genes in tartary buckwheat based on the whole genome.

**Results:**

In this study, 96 *FtbZIP* genes named from *FtbZIP1* to *FtbZIP96* were identified and divided into 11 subfamilies according to their genetic relationship with 70 *bZIPs* of *A. thaliana*. *FtbZIP* genes are not evenly distributed on the chromosomes, and we found tandem and segmental duplication events of *FtbZIP* genes on 8 tartary buckwheat chromosomes. According to the results of gene and motif composition, *FtbZIP* located in the same group contained analogous intron/exon organizations and motif composition. By qRT-PCR, we quantified the expression of *FtbZIP* members in stem, root, leaf, fruit, and flower and during fruit development. Exogenous ABA treatment increased the weight of tartary buckwheat fruit and changed the expressions of *FtbZIP* genes in group A.

**Conclusions:**

Through our study, we identified 96 *FtbZIP* genes in tartary buckwheat and synthetically further analyzed the structure composition, evolution analysis and expression pattern of *FtbZIP* proteins. The expression pattern indicates that *FtbZIP* is important in the course of plant growth and development of tartary buckwheat. Through comprehensively analyzing fruit weight and *FtbZIP* genes expression after ABA treatment and endogenous ABA content of tartary buckwheat fruit, ABA may regulate downstream gene expression by regulating the expression of *FtPinG0003523300.01* and *FtPinG0003196200.01*, thus indirectly affecting the fruit development of tartary buckwheat. This will help us to further study the function of *FtbZIP* genes in the tartary buckwheat growth and improve the fruit of tartary buckwheat.

**Electronic supplementary material:**

The online version of this article (10.1186/s12864-019-5882-z) contains supplementary material, which is available to authorized users.

## Background

Transcription factors (TFs) are key regulatory factors in many signaling networks and are sequence-specific binding proteins that bind to a target gene promoter to regulate its transcription and thus respond to plant growth and development. The TFs encoded by the eukaryote genome accounts for 3.5–7.0% of the total estimated genome [1]. These TFs can be divided into 40–60 families according to the similarity of amino acid sequences and the structure of conserved domains [2, 3]. Among them, the basic leucine zipper (bZIP) contains a highly conserved *bZIP* domain and is widely distributed in eukaryotes [4, 5]. The bZIP domain is between 60 and 80 amino acids and contains two functional domains, a base region and a leucine zipper region [6]. The base region is highly conserved, located at the N end of the region and composed of a conserved motif that binds to DNA and is involved in subcellular localization. The leucine zipper region is variable and is a repeated sequence composed of leucine or other hydrophobic amino acids; 9 of these amino acids are located at the C-terminus, creating an amphipathic helix [7–10]. To date, *bZIP* has been widely identified and analyzed in many plants, including *Arabidopsis thaliana* (*A. thaliana*), *Oryza sativa* (rice), *Solanum lycopersicum* (tomato)*, Zea mays* (maize)*, Sorghum bicolor* (sorghum)*, Fragaria ananassa* (strawberry)*, Daucus carota* (carrot)*, Hordeum vulgare* (barley)*, Cucumis sativus* (cucumber) and *Ricinus communis* (castor bean) [4, 9–18]. It was found that *bZIP* is involved in many important biological activities, such as cell elongation [19, 20], histological differentiation [21–23], metabolic activity [24], seed storage protein gene regulation, as well as embryogenesis and seed maturation [25]. At the same time, the *bZIP* is involved in the response to abiotic and biotic stresses, including hormone and sugar signaling [26, 27], photoreaction [28, 29], salt and drought tolerance [30, 31], and pathogen defense [32, 33]. In these processes (signal transduction, stress response and development), the expression of *bZIP* gene family is mainly regulated by abscisic acid (ABA). ABA is a significant plant hormone that plays roles in regulating gene expression and related physiological processes in abiotic stress response [9, 34]. ABA responsive element (ABRE) is the main cis-acting element that regulates response of the ABA gene. ABF/AREB/ABI5 in *A. thaliana bZIP* group A, which are ABRE binding TFs, are directly associated with the ABA response and have been identified to play a significant role in the regulation of ABA [35]. ABA Insensitive 5 (*ABI5*) plays a major role in activating plant ABA signaling [36, 37]. Starch is the main storage material of tartary buckwheat seeds, accounting for more than 86% of nutrients [38]. There is coexpression of some starch metabolism-related enzyme genes and ABA induced genes [39].

*Fagopyum talaricum* (tartary buckwheat) (2*n* = 8) that belongs to the Polygonaceae family is an important food for health and nutrition so that it has now been introduced in many countries [40]. The rutin in tartary buckwheat can reduce hypertension and arteriosclerosis in addition to exhibiting antioxidant activity, and quercetin shows antimicrobial activity [41–43]. Therefore, tartary buckwheat is also a valuable medicinal plant. Tartary buckwheat contains more quercetin and 30 to 150 times more rutin than common buckwheat [44]. Tartary buckwheat also contains crude fiber, vitamins B1, B2, and B6 as well as valuable proteins consisting of balanced amino acids [45, 46]. Although the *bZIP* family has been widely studied in many plants at the whole genome level, the study of *bZIP* in tartary buckwheat is still lacking [47, 48]. Because the *bZIP* gene has many important physiological functions and is important to the plant, it is necessary to systematically study the *Fagopyum talaricum bZIP (FtbZIP)* family. A recent study reported the genomic sequence of tartary buckwheat, laying a foundation for studying the characteristics, evolution and expression of genome-wide *FtbZIP* [49]. In this study, 96 *FtbZIP* genes (*FtbZIPs*) were identified and divided into 11 subfamilies through the phylogenetic analysis of tartary buckwheat and *A. thaliana*. We performed detailed analyses of *FtbZIPs*, including gene structure, motif composition, chromosomal distribution and gene duplication. In addition, we constructed a typical comparative system diagram between tartary buckwheat and other dicotyledonous/monocotyledonous plants, including *A. thaliana*, rice, *Beta vulgaris* (beet), *Glycine max* (soybean), tomato, *Vitis vinifera* (grape) and *Helianthus annuus* (sunflower). In addition, the expression patterns of 20 selected *FtbZIP* genes in diverse tissues/organs (stem, root, leaf, flower and fruit) were also analyzed using Real-time Quantitative Polymerase Chain Reaction (qRT-PCR). We determined the differential expression profiles of these 20 *FtbZIP* genes at different fruit development stages. Further, the tartary buckwheat fruit weight and the expression of related *FtbZIPs* were analyzed under the treatment of exogenous ABA. Two *FtbZIP* genes, *FtPinG0003523300.01* and *FtPinG0003196200.01,* were selected from group A, and their relationship and function with ABA were analyzed. This study provides an opportunity to further study the functions of *FtbZIP* genes throughout tartary buckwheat development stages and crop improvement.

## Results

### Identification of the *FtbZIP* genes in tartary buckwheat

To identify the *bZIP* genes of tartary buckwheat, we used two BLAST methods to identify all possible *bZIP* members that were 96 *bZIP* genes in the tartary buckwheat genome (Additional file 1: Table S1). In this study, according to the top-to-bottom position of the *bZIP* genes on the chromosomes, the *FtbZIP* genes were named *FtbZIP1* to *FtbZIP96*.

We provided the gene characteristics including coding sequence length (CDS), molecular weight (MW), isoelectric point (PI) and subcellular localization (Additional file 1: Table S1). The 96 predicted FtbZIP proteins ranged from 68 *(FtPinG0005198100.01)* to 495 amino acids (aa) *(FtPinG0003081400.01)*, with an average of 271 aa (Additional file 1: Table S1). The MW of the proteins ranged from 7.73 *(FtPinG0005198100.01)* to 54.31 *(FtPinG0007266400.01)* KDa, with an average of 30.16 KDa. And the PI varied from 4.85 (*FtPinG0001659300.01*) to 11.41 (*FtPinG0009370700.01),* with an average of 7.66. The subcellular localization showed that 94 *FtbZIPs* were situated in the nucleus, while 2 *FtbZIPs* were situated in the mitochondrion.

### Phylogenetic analysis and classification of *FtbZIP* genes

**Fig. 1:**
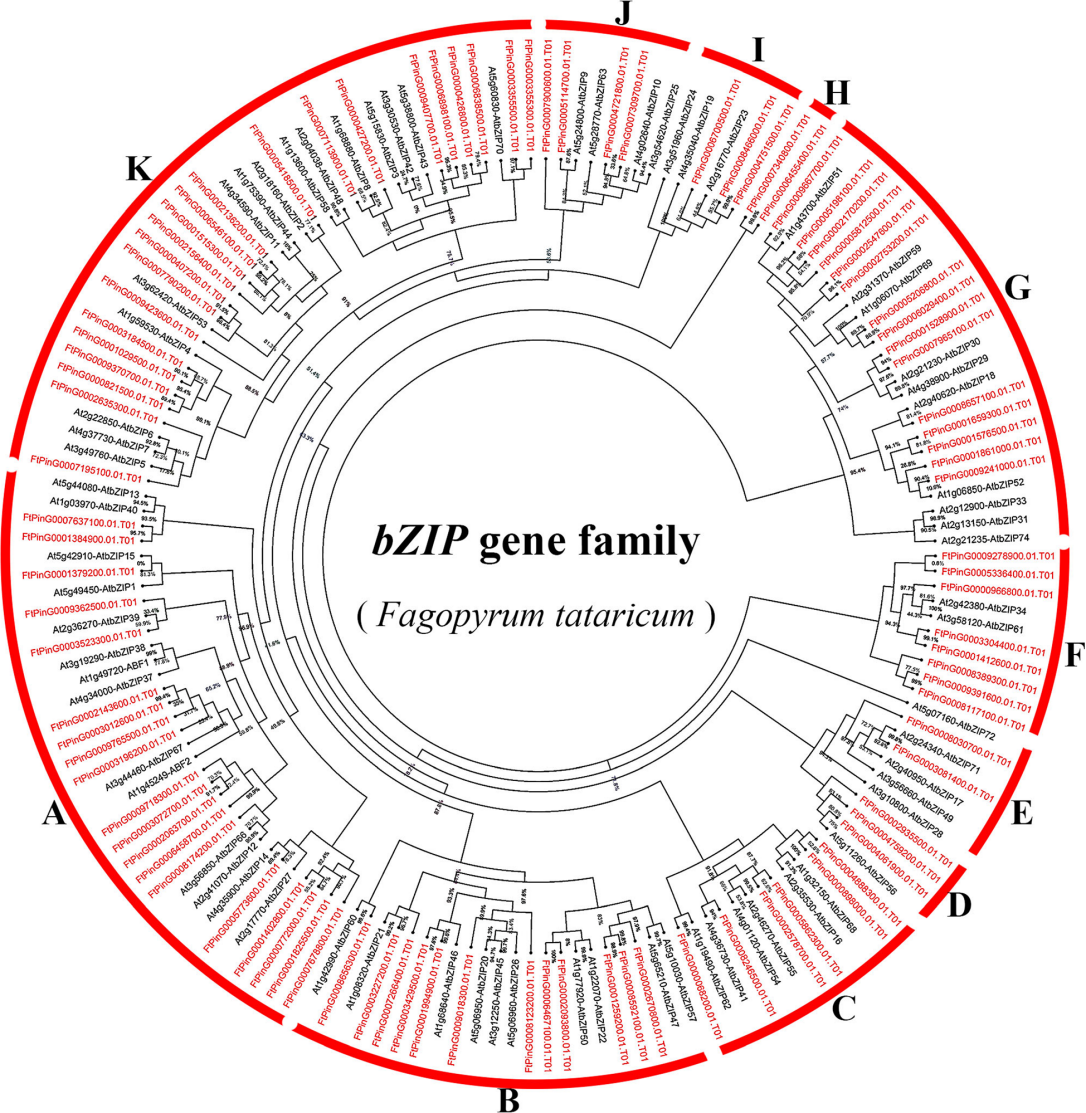
Unrooted phylogenetic tree representing the relationships among 96 *FtbZIP* genes from tartary buckwheat and 70 *AtbZIP* genes from *A. thaliana*. 96 *FtbZIP* genes and 70 *AtbZIP* genes are classified into group **a**, **b**, **c**, **d**, **e**, **f**, **g**, **h**, **i**, **j** and **k**. Group H represents the *FtbZIP* genes that cannot be classified. The *bZIP* genes from tartary buckwheat and *A. thaliana* are marked in red and black, respectively. Percentages beside all branches are bootstrap support values generated from 1000 replicates

The previous study identified 75 *bZIP* genes in the classical research crop *A. thaliana* [10]. In order to study the evolutionary relationship between tartary buckwheat *bZIP* and *A. thaliana bZIP* (*AtbZIP*) members, we constructed an unrooted Maximum Likelihood (ML) tree with 70 *AtbZIPs* and 96 *FtbZIPs* (Fig. 1). As shown in Fig. 1a total of 166 *bZIPs* from tartary buckwheat and *A. thaliana* were separated into 11 groups (A to K) based on the classification in *A. thaliana* [10]. Group K was the biggest group, containing 22 genes of the *FtbZIP* family. Both groups E and H were the smallest clusters, each including 2 members. Group H was not homologous to *AtbZIP*.

### Gene structure and conserved motif compositions of *FtbZIPs* gene family

**Fig. 2:**
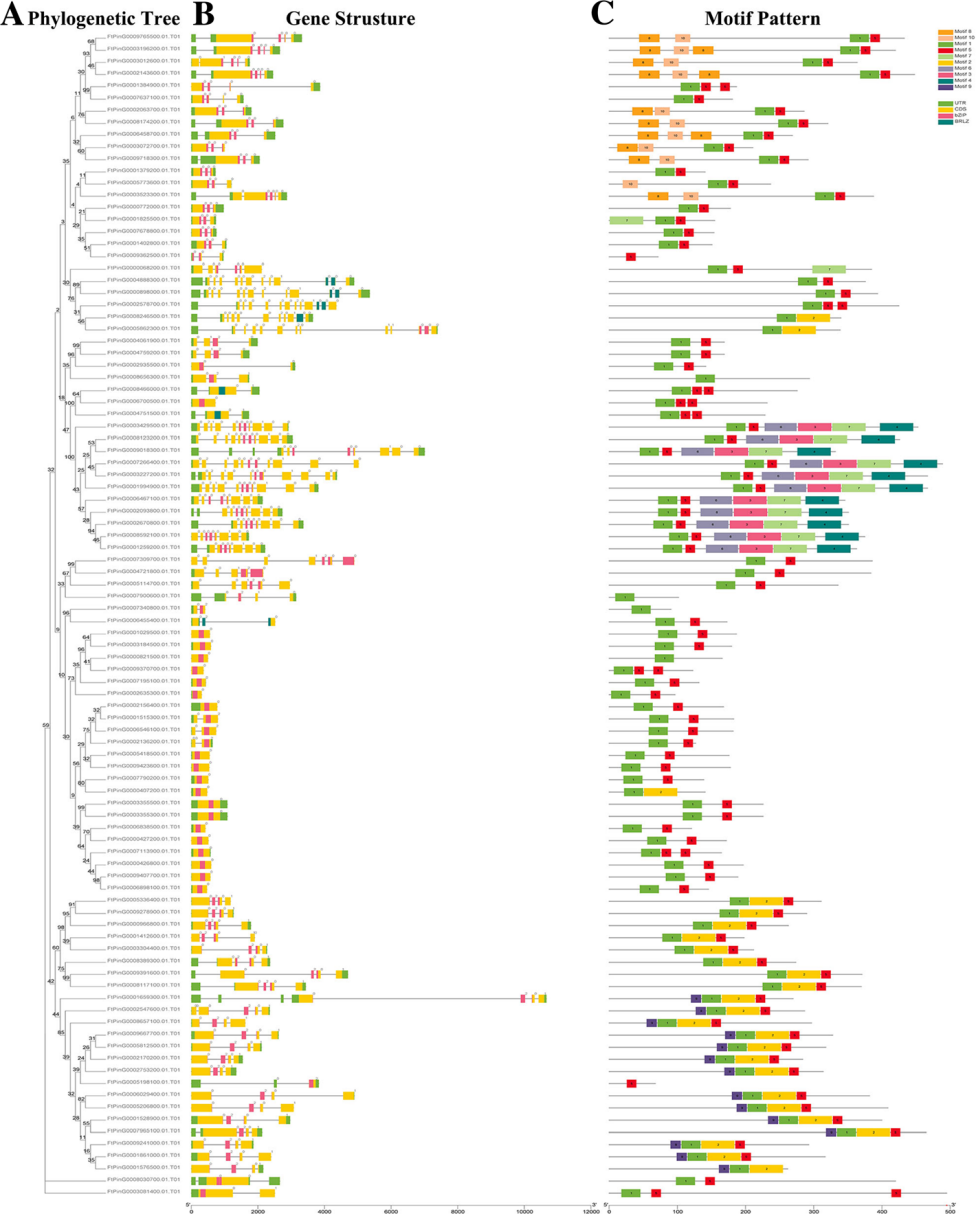
Phylogenetic relationships, gene structures and compositions of the conserved protein motifs of the *FtbZIP* genes from tartary buckwheat. **a** The phylogenetic tree was constructed based on the full-length sequences of tartary buckwheat bZIP proteins. **b** Exon-intron structures of the tartary buckwheat *bZIP* genes. Green boxes indicate untranslated 5′- and 3′-regions; yellow boxes indicate exons; and black lines indicate introns. The bZIP domain is highlighted by a pink box. The number indicates the phases of the corresponding introns. **c** The motif compositions of the tartary buckwheat bZIP proteins. The motifs, numbered 1–10, are displayed in different colored boxes. The sequence information for each motif is provided in Additional file 2: Table S2. The protein length can be estimated using the scale at the bottom

To study the components of the *FtbZIP* gene structure, we examined the exons and introns, including their amount and distribution among *FtbZIP* genes (Fig. 2b). In general, the exon/intron structures of most *FtbZIP* genes from the same group have a similar exon/intron number. The results showed that 22 (22.9%) of the 96 *FtbZIP* genes had no introns, most of which were concentrated in group K. Among the other *FtbZIP* genes containing introns, the number of introns ranged from 1 to 11. The number of introns in the genes of the same group varied only slightly, mostly from 0 to 4. The number of exons varied from 1 to 10, demonstrating that there were some differences in degree among the 96 *FtbZIP* genes. The position of the exons in each subgroup was diverse. However, the exons from the same subgroup were typically similar in length and number.

To research the specific parts of the FtbZIP proteins, the 10 conserved motifs shown in Fig. 2c were studied. It can be observed that most FtbZIP proteins in the same clade had the same motif constitutions that further confirmed the grouping results (Additional file 2: Table S2). For example, all members of the subfamilies D, E and I only have motifs 1 and 5; group F shares motifs 1, 2 and 5; group C and J contain motif 1; group G possesses motifs 1, 2, 5 and 9, except for FtPinG0005198100.01 and FtPinG0001576500.01; and group B possesses motifs 1, 3, 4, 5, 6 and 7 except FtPinG0008656300.01. Motifs 1 and 5 are widely present in most (89.6%) FtbZIP proteins. However, motifs 8 and 10 were only found in group A; motif 4 only existed in group B; motif 9 was only found in group G. In group K, only FtPinG0000407200.01 had motif 2.

### Chromosomal distribution, gene duplication events and synteny analysis of *FtbZIP* genes

**Fig. 3:**
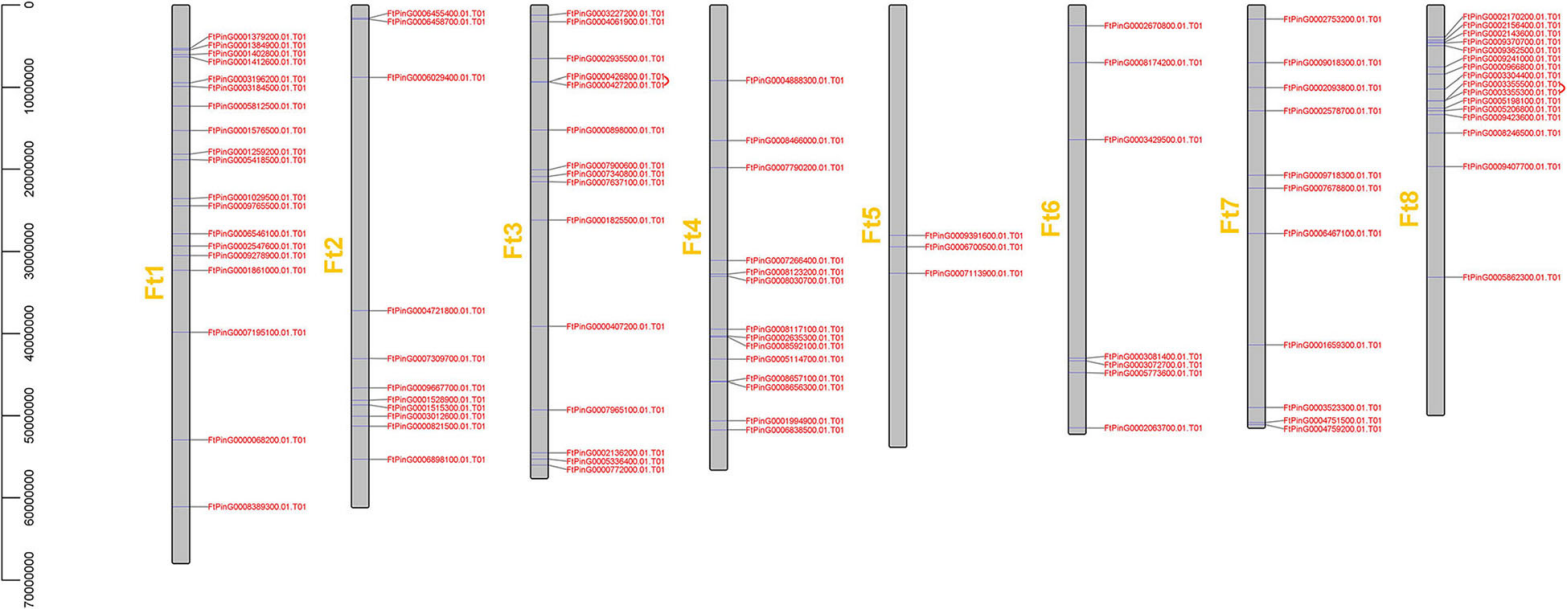
Schematic representations of the chromosomal distribution of the tartary buckwheat *bZIP* genes. The chromosome number is indicated to the left of each chromosome

**Fig. 4:**
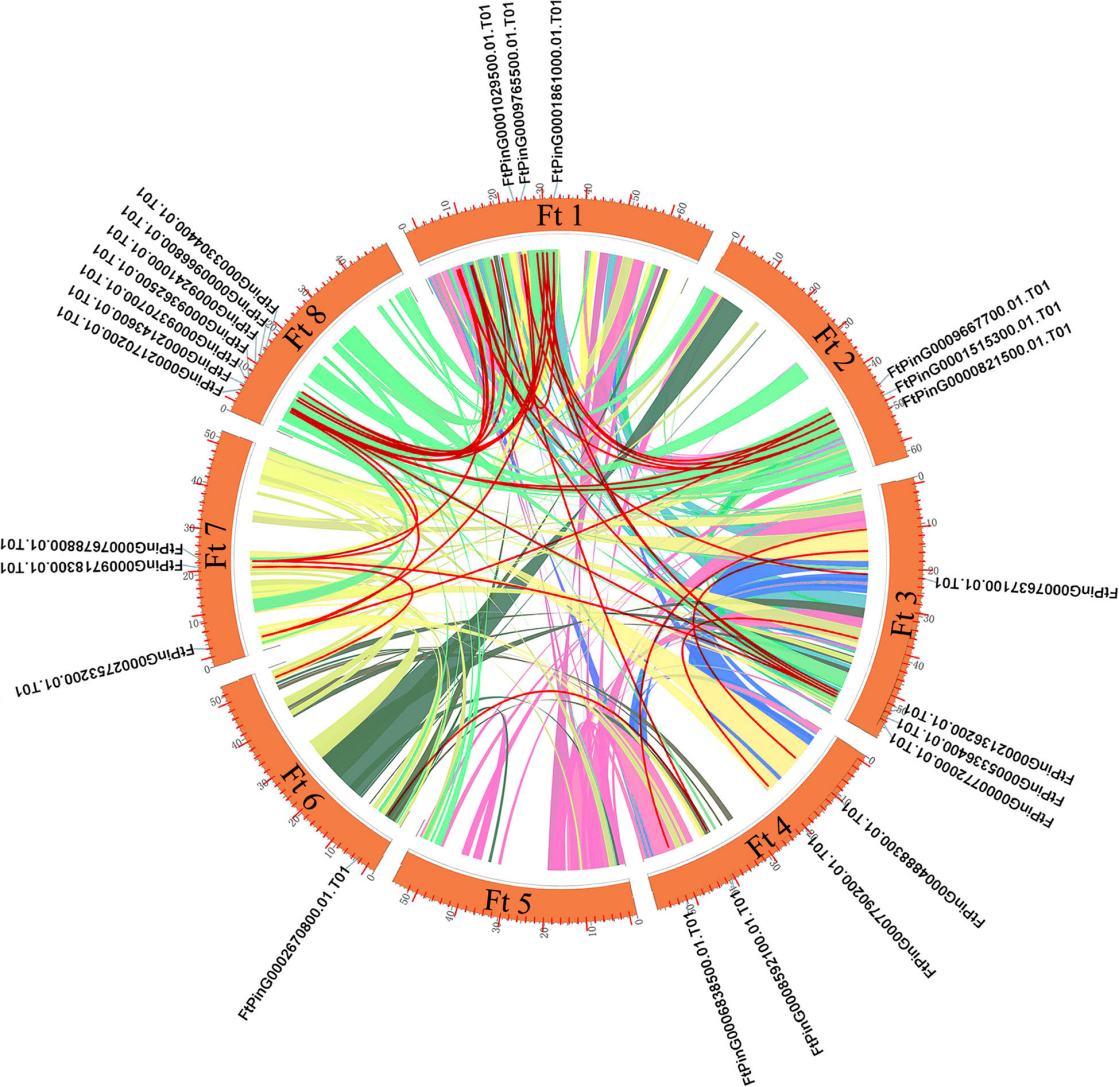
Schematic representations of the interchromosomal relationships of the tartary buckwheat *bZIP* genes. Colored lines indicate all syntenic blocks in the tartary buckwheat genome

As shown in Fig. 3, we found that 96 *FtbZIP* genes were dispersed on 8 tartary buckwheat linkage groups (LGs). Specific regions had a relatively high density of *FtbZIP* genes. LG1 had the most *FtbZIP* genes (19), and LG5 had the fewest *FtbZIP* genes (3). To study the evolutionary regulation of the *FtbZIP* gene family, we described the gene duplication events, including tandem and segmental duplication events [50]. From the results, we know that the duplications of the *FtbZIP* gene family included both tandem and segmental duplication. As shown in Fig. 3, two pairs of *FtbZIP* genes (*FtPinG0000426800.01* and *FtPinG0000427200.01, FtPinG0003355500.01* and *FtPinG0003355300.01*) and two tandem repeat regions were located on tartary buckwheat chromosomes 3 and 8, respectively. Furthermore, in 8 LGs of the tartary buckwheat genome, there are many groups of repeated *FtbZIP* gene fragments located on 8 tartary buckwheat chromosomes, and all of them are located on two different LGs (Fig. 4).

### Evolutionary analyses of *FtbZIP* genes and several other species

**Fig. 5:**
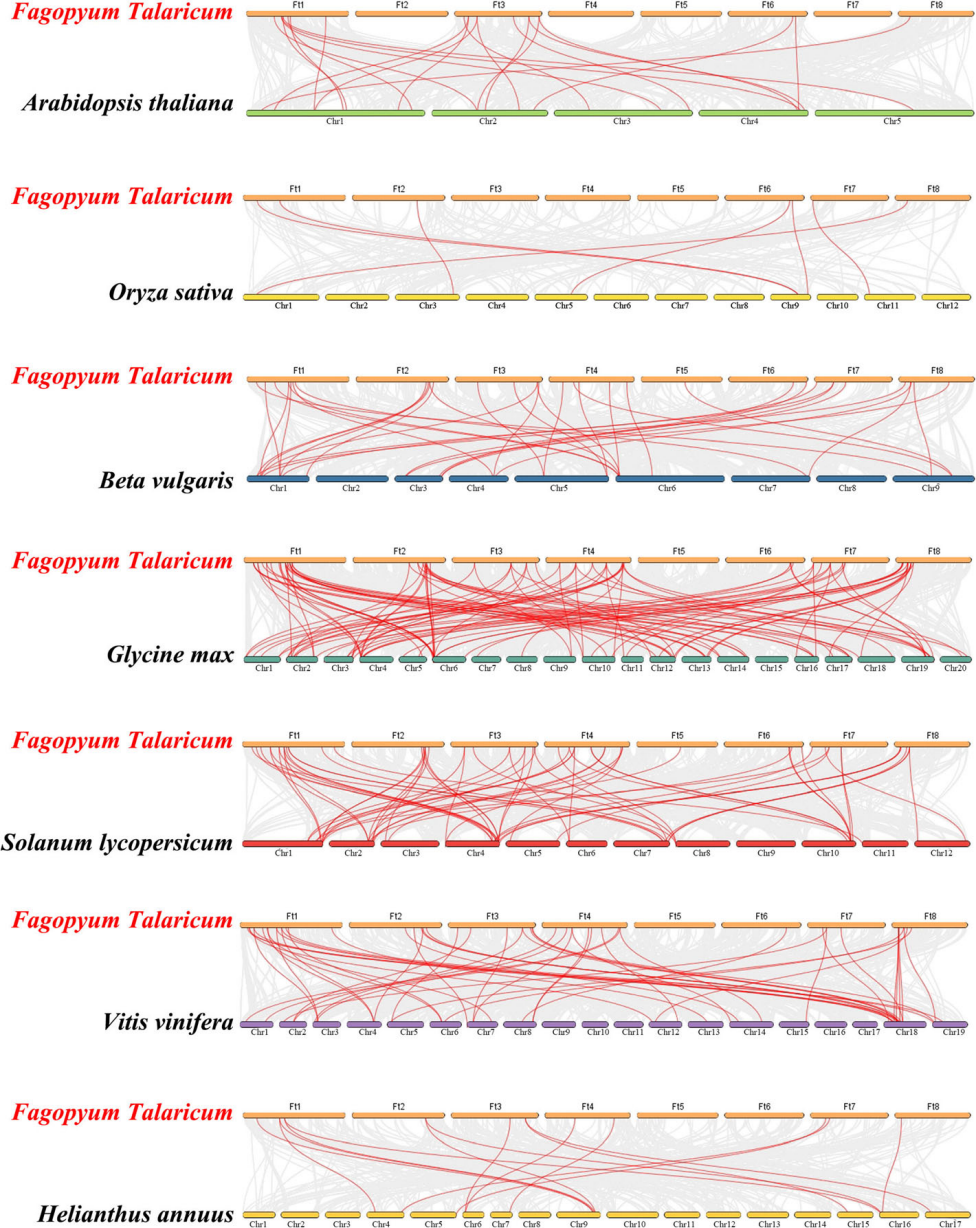
Synteny analyses between the *bZIP* genes of tartary buckwheat and those of seven representative plant species

To explore the phylogenetic mechanisms of the tartary buckwheat *bZIP* family, we constructed seven typical comparative system diagrams comparing tartary buckwheat and six other dicotyledonous plants (*A. thaliana*, beet, soybean, tomato, grape and sunflower) and one monocotyledonous plant (rice), as shown in Fig. 5. In total, *FtbZIP* genes displayed syntenic relationship in different degrees with soybean (102), then tomato (64), grape (52), beet (35), *A. thaliana* (21), sunflower (15) and rice (7) (Additional file 3: Table S3). The result indicated that tartary buckwheat *bZIP* genes relatively had a more similar relationship with soybean *bZIP* genes. The *bZIP* genes might have evolved from the common ancestor in different plants.

**Fig. 6:**
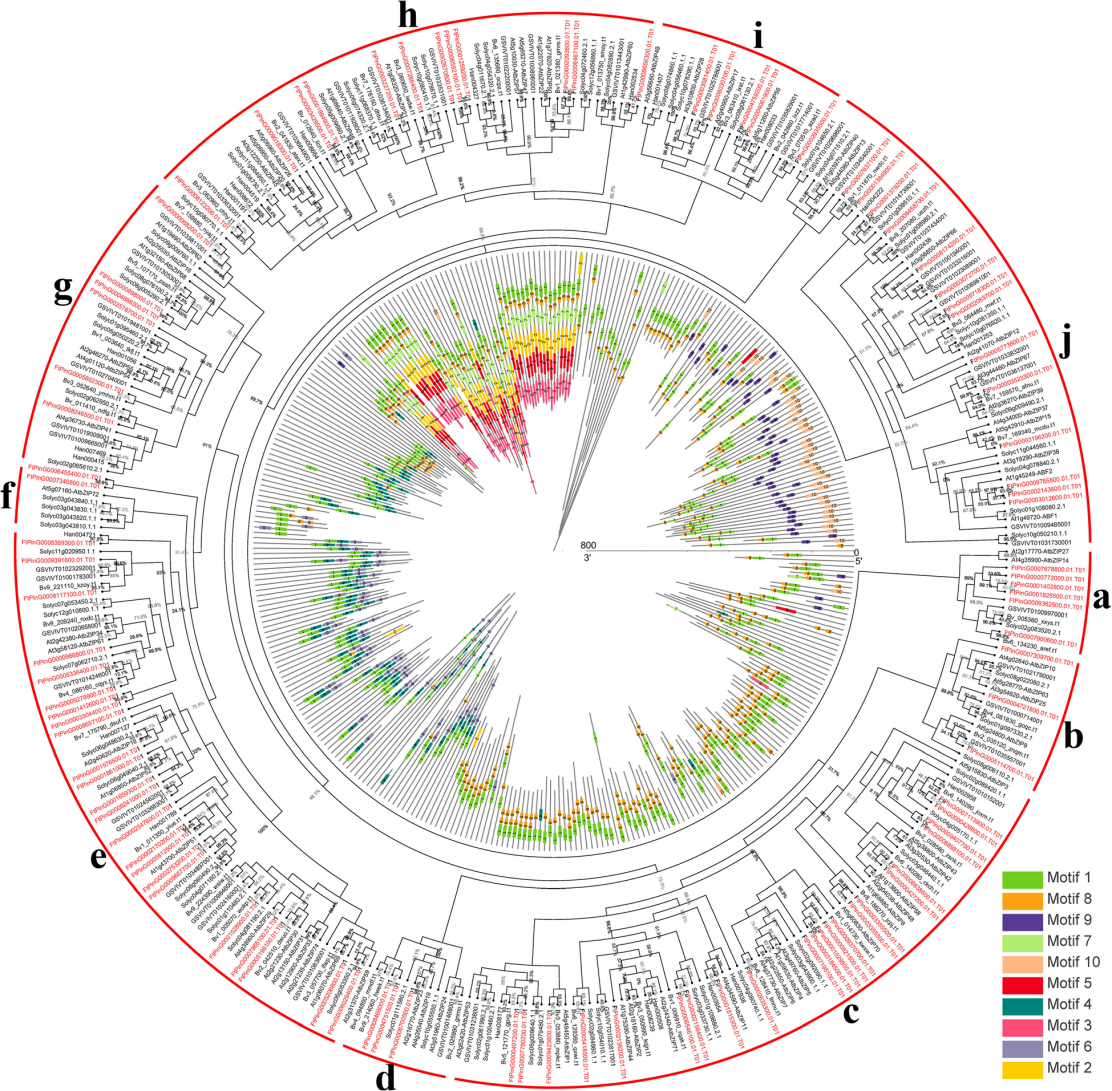
Phylogenetic relationships and motif compositions of the bZIP proteins from six different plant species (tartary buckwheat, *A. thaliana*, beet, tomato, grape and sunflower). The phylogenetic tree includes 96 *bZIP* genes from tartary buckwheat, 70 from *A. thaliana*, 48 from beet, 70 from tomato, 47 from grape and 23 from sunflower. *bZIP* genes from multiple species are classified into group **a**, **b**, **c**, **d**, **e**, **f**, **g**, **h**, **i** and **j**. The *bZIP* genes from tartary buckwheat and other plant species are marked in red and black, respectively. Percentages beside all branches are bootstrap support values generated from 1000 replicates. The motifs, numbered 1–10, are displayed in different colored boxes. The sequence information for each motif is provided in Additional file 2: Table S2

For the sake of studying the evolutionary relationship of the *bZIP* family genes among tartary buckwheat and five other plants (*A. thaliana*, beet, tomato, grape and sunflower), we constructed an unrooted ML tree according to the protein sequences of the 96 *FtbZIP* genes and five other plant *bZIP* genes (Fig. 6). The *bZIP* genes were divided into 10 clades (from *a* to *j*). The clade *e* had the most *FtbZIP* genes, while group *f* had only 2 *FtbZIP*. Using MEME web servers, we selected the conservative motifs shared among the bZIP proteins (Additional file 2: Table S2). In total, 10 conservative motifs were analyzed among these clades, as shown in Fig. 6. Almost all the members of every clade have motif 1. Additionally, motifs 1 and 8 widely exist in most clades. However, clades *c, d, e, f, g* and *j* have no motif 7. Most bZIP members in the same group have common motifs. For example, most *bZIP* genes of the clade *h* share motifs 1, 2, 3, 5, 7 and 8, indicating potential functional similarities among the bZIP proteins.

### Expression patterns of *FtbZIP* genes in different plant tissues

**Fig. 7:**
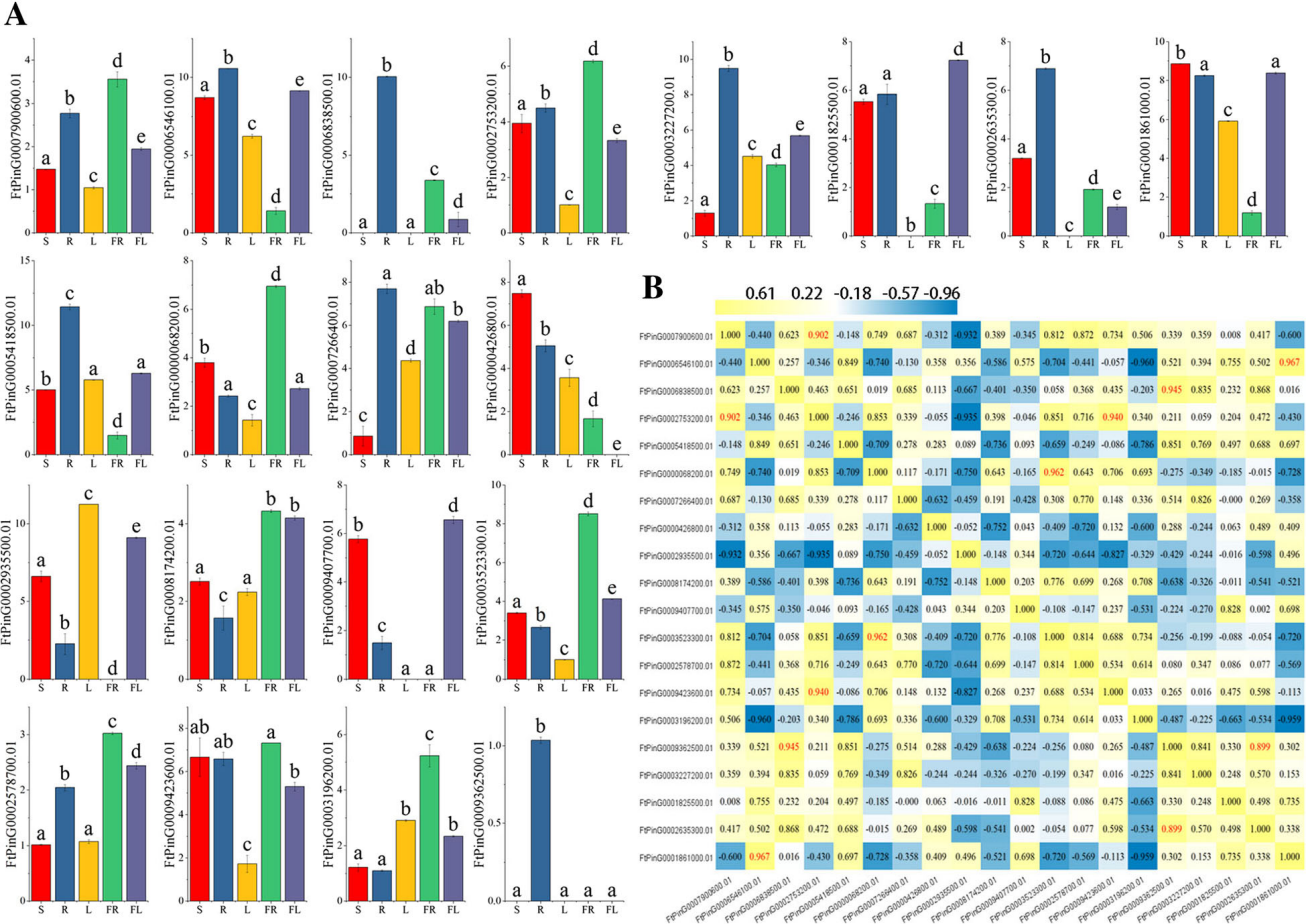
Tissue-specific gene expression of 20 tartary buckwheat *bZIP* genes and the correlations between the gene expression patterns of *FtbZIPs*. **a** The expression patterns of 20 tartary buckwheat *bZIP* genes in stem (S), root (R), leaf (L), fruit (FR) and flower (FL) tissues were examined by qPCR. The error bars were obtained from three measurements. Lowercase letter(s) above the bars indicate significant differences (α = 0.05, LSD) among the treatments. **b** A positive number indicates a positive correlation; a negative number indicates a negative correlation. The red numbers indicate a significant correlation at the 0.05 level

In angiosperms, increasing studies have suggested that *bZIP* genes widely play an important role in the process of growing and developing [51]. To gain insight into the physiological role of the *FtbZIP* gene, we used qRT-PCR to determine the expression of the *FtbZIP* gene family members throughout growth and development. The expression patterns of 20 *FtbZIP* genes in stem, root, leaf, fruit and flower were analyzed (Fig. 7a). The histograms exhibited that the transcript abundance of 20 *FtbZIP* genes was significantly different among the various organs and tissues, indicating that *FtbZIP* had various functions in the developmental stages of tartary buckwheat plant. This study found that some *FtbZIP* genes had specific expression patterns in specific organs/tissues in tartary buckwheat. For instance, seven *FtbZIP* genes (*FtPinG0006546100.01, FtPinG0006838500.01, FtPinG0003227200.01, FtPinG0002635300.01, FtPinG0005418500.01, FtPinG0007266400.01* and *FtPinG0009362500.01*) were more highly expressed in root than the other organs/tissues; interestingly, *FtPinG0009362500.01* was expressed only in the root (Fig. 7a). *FtPinG0002935500.01* highly expressed in tartary buckwheat leaf and two *FtbZIP* genes (*FtPinG0001825500.01* and *FtPinG0009407700.01*) exhibited high expression in tartary buckwheat flower. And eight *FtbZIP* genes (*FtPinG0007900600.01, FtPinG0002753200.01, FtPinG0000068200.01, FtPinG0008174200.01, FtPinG0003523300.01, FtPinG0002578700.01, FtPinG0009423600.01* and *FtPinG0003196200.01)* showed relatively high expression in fruit.

Additionally, the correlation of the expressions of these 20 *FtbZIP* genes was analyzed (Fig. 7b). A majority of the *FtbZIP* genes were positively correlated, and several pairs of *FtbZIP* genes (*FtPinG0002753200.01* and *FtPinG0007900600.01/FtPinG0009423600.01; FtPinG0000068200.01* and *FtPinG0003523300.01; FtPinG0009362500.01* and *FtPinG0006838500.01/ FtPinG0002635300.01; FtPinG0001861000.01* and *FtPinG0006546100.01*) were significantly correlated (Fig. 7b).

### Differential expression of *FtbZIP* genes during the fruit development of tartary buckwheat

**Fig. 8:**
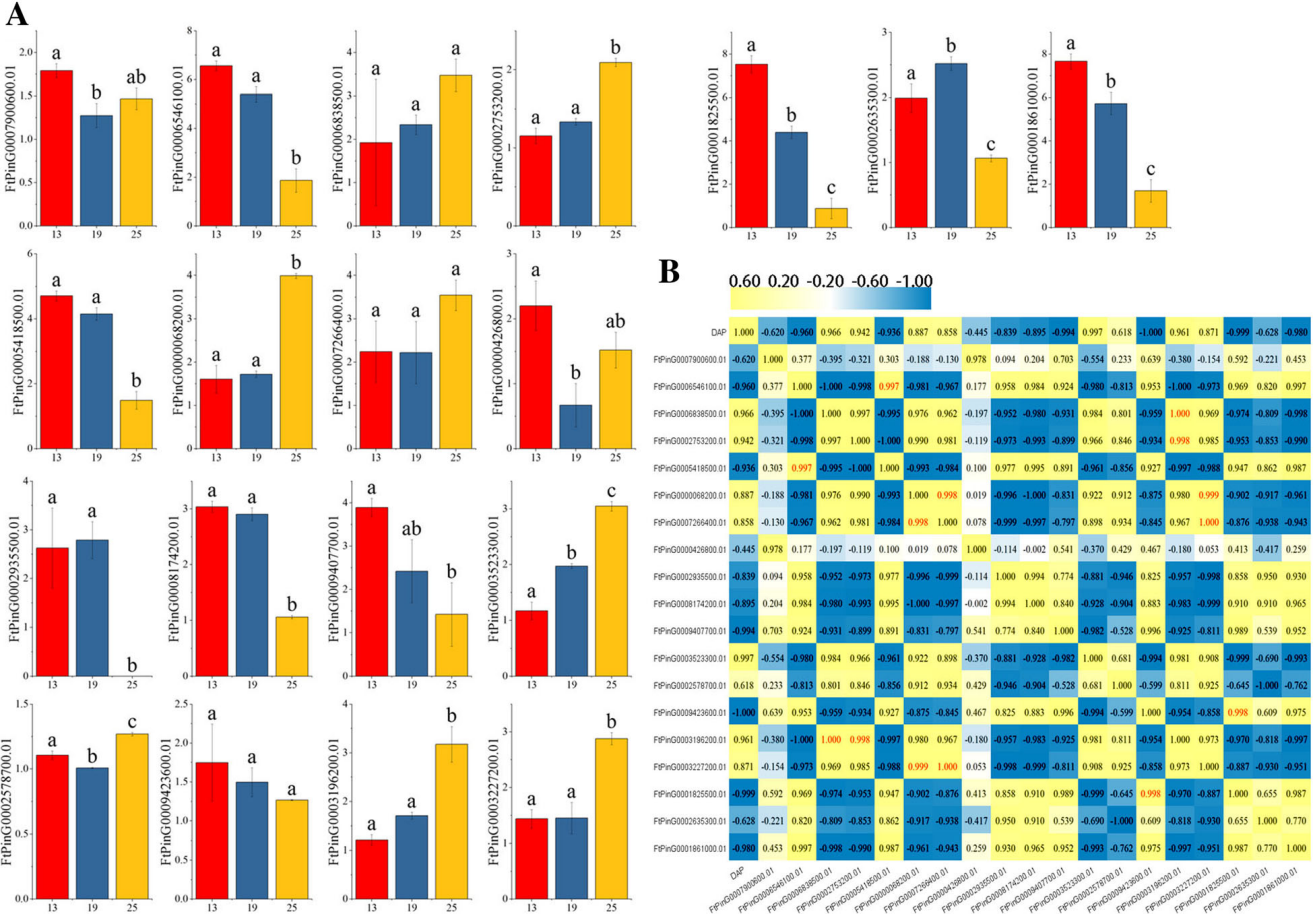
Gene expression levels of 19 tartary buckwheat *bZIP* genes during fruit development and the correlations of the *FtbZIP* gene expression patterns during fruit development. **a** The expression patterns of 19 tartary buckwheat *bZIP* genes in the fruit development stage were examined using a qPCR assay. The error bars were obtained from three measurements. Lowercase letter(s) above the bars indicate significant differences (α = 0.05, LSD) among the treatments. **b** A positive number indicates a positive correlation; a negative number indicates a negative correlation. Red numbers indicate a significant correlation at the 0.05 level

The expression of the transcriptional products of 19 *FtbZIP* genes (*FtPinG0009362500.01* had no expression in tartary buckwheat fruit) throughout the growth and development of tartary buckwheat fruit was analyzed (Fig. 8a). The histograms exhibited that the expressed products abundance of 19 *FtbZIP* genes was markedly diverse at 13 (green fruit stage), 19 (expansion stage), and 25 (discoloration stage) days after pollination (DAP) [38], indicating that *FtbZIP* genes have numerous functions in tartary buckwheat fruit development. For example, the expression levels of seven *FtbZIP* genes (*FtPinG0006838500.01*, *FtPinG0002753200.01*, *FtPinG0000068200.01, FtPinG0007266400.01*, *FtPinG0003523300.01*, *FtPinG0003196200.01* and *FtPinG0003227200.01*) increased progressively throughout tartary buckwheat fruit development, as shown in Fig. 8a. The expression of seven *FtbZIP* genes (*FtPinG0006546100.01, FtPinG0001825500.01, FtPinG0001861000.01, FtPinG0005418500.01, FtPinG0008174200.01, FtPinG0009407700.01* and *FtPinG0009423600.01)* decreased by varying degrees. The expression of five other genes fluctuated. Overall, 9 (47.4%), 8 (42.1%) and 2 (10.5%) *FtbZIP* genes in tartary buckwheat fruit showed relatively high expression at green fruit stage, discoloration stage and expansion stage, respectively.

Additionally, *FtPinG0009423600.01/FtPinG0001825500.01* had a significant negative correlation with fruit development. The correlation of the expressions of these 19 *FtbZIP* genes was analyzed in tartary buckwheat fruit. Most of the *FtbZIP* genes were positively correlated, and the *FtbZIP* genes (*FtPinG0006546100.01* and *FtPinG0005418500.01; FtPinG0000068200.01* and *FtPinG0007266400.01; FtPinG0009423600.01* and *FtPinG0001825500.01; FtPinG0003196200.01* and *FtPinG0006838500.01/FtPinG0002753200.01; FtPinG0003227200* and *FtPinG0000068200.01/FtPinG0007266400.01)* were significantly correlated (Fig. 8b).

### The fruit weight and expression of the group A *FtbZIP* genes at 25 DAP under exogenous ABA treatment conditions

**Fig. 9:**
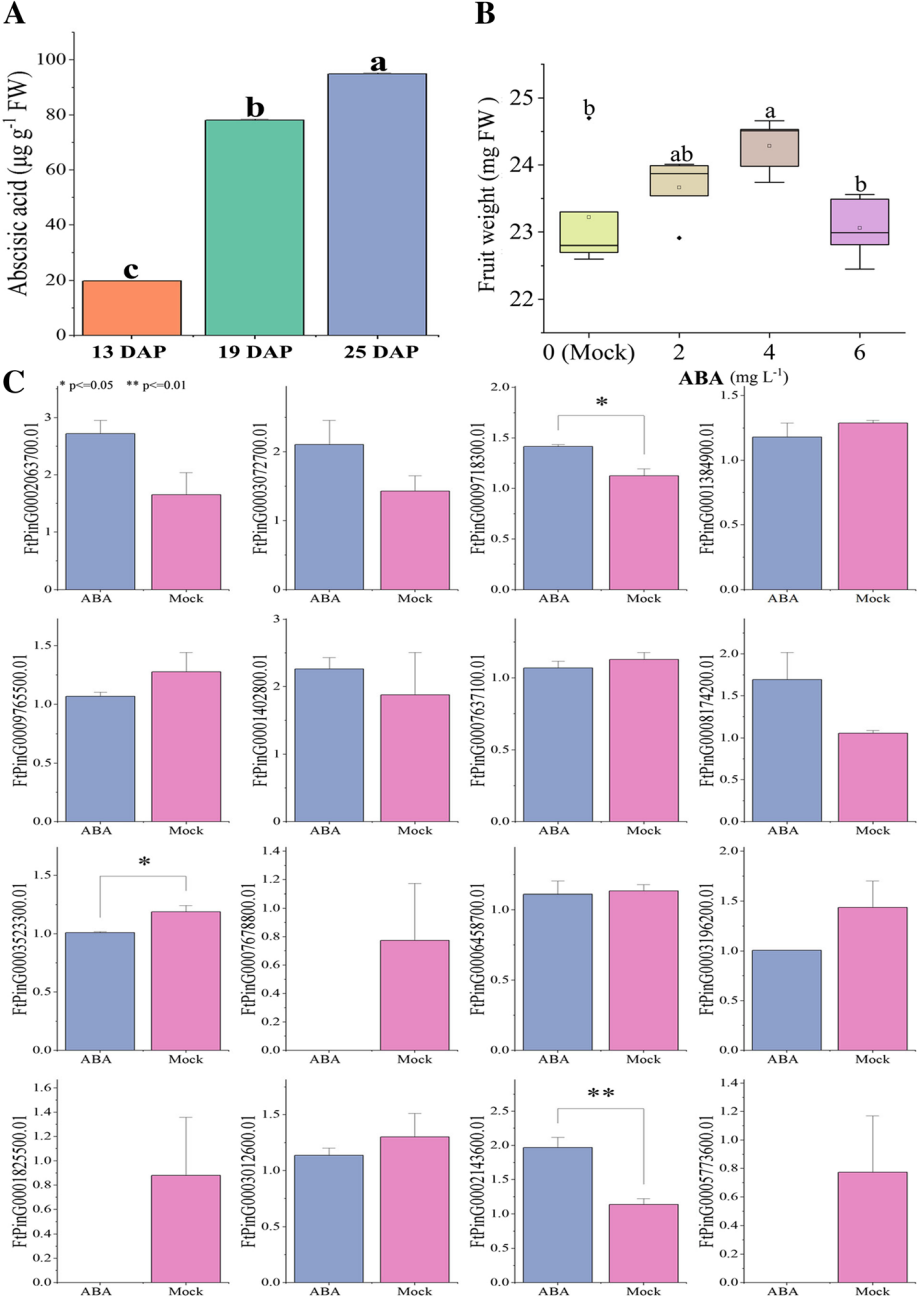
Endogenous ABA content in tartary buckwheat fruits and changes in fruits weight and gene expression after exogenous ABA treatment. Endogenous ABA content throughout fruit development. **b** Weight of the fruits treated with different concentrations of exogenous ABA. X-axis: concentration of ABA treatment, y-axis: weight of mature fruit. Error bars were obtained from five measurements. **c** Expression of *FtbZIP* genes from group **a** at 25 DAP treated with 4 mg L^− 1^ exogenous ABA. Mock: the same amount of water treatment. Small letter(s) above the bars indicate significant differences (α = 0.05, LSD) among the treatments * and ** indicate significant correlations at the 0.05 and 0.01 levels, respectively

According to the background mentioned, ABA induces the expression of *bZIP* genes during growth and development, and ABA is related to genes encoding starch metabolism enzyme. Only group A of the *AtbZIP* genes were directly related to the ABA responses, and genes from tartary buckwheat group A are homologous to those from *A. thaliana* group A. Therefore, the plant was treated with exogenous ABA, and the expression of group A *FtbZIP* genes in the fruit at 25 DAP was measured. Previous studies showed that there is a certain relationship between the size of tartary buckwheat fruit and ABA [38, 52]. We observed changes in the endogenous ABA content of tartary buckwheat fruit during fruit development (13 DAP to 25 DAP). The ABA content increased throughout the stages shown in Fig. 9a. In addition, ABA increased significantly from 13 DAP to 19 DAP. To further study the response of group A *FtbZIP* genes to ABA, we sprayed the whole tartary buckwheat plant with different concentrations of ABA (2, 4 or 6 mg L^− 1^) at the bud stage. In contrast, with the blank treatment group, the tartary buckwheat fruit weight increased to varying degrees under ABA treatment and significantly increased under the treatment of 4 mg L^− 1^ ABA (Fig. 9b). Thus, 4 mg L^− 1^ ABA was selected as the optimal treatment to increase the fruit weight of tartary buckwheat. The *FtbZIPs* from group A were homologous to the *AtbZIPs* from group A (Fig. 1). We detected the expression of the group A *FtbZIP* genes in tartary buckwheat fruit at the discoloration stage (25 DAP) with 4 mg L^− 1^ exogenous ABA treatment. We noticed that the expression of most *FtbZIP* genes varied remarkably after ABA treatment compared with the blank group from the data shown in Fig. 9c. At the discoloration stage, the expression of *FtPinG0002063700.01, FtPinG0003072700.01, FtPinG0009718300.01, FtPinG0001402800.01, FtPinG0008174200.01* and *FtPinG0002143600.01* significantly increased after ABA treatment. However, the expression of *FtPinG0001384900.01, FtPinG0009765500.01, FtPinG0003523300.01, FtPinG0007678800.01, FtPinG0003196200.01, FtPinG0001825500.01, FtPinG0003012600.01* and *FtPinG0005773600.01* decreased after ABA treatment; especially *FtPinG0007678800.01, FtPinG0001825500.01* and *FtPinG0005773600.01,* which were not expressed after ABA treatment at 25 DAP. There was little difference in the expression of *FtPinG0007637100.01* and *FtPinG0006458700.01* before and after ABA treatment. In general, exogenous ABA treatment caused an increase of fruit weight of tartary buckwheat and influenced the expression rates of analyzed genes at 25 DAP.

## Discussion

Tartary buckwheat is an important cash crop [53]. The *bZIP* gene family has been reported to have abundant functions in a variety of biological processes [10]. However, research on the whole genome of the *bZIP* gene family in tartary buckwheat is relatively lacking. Zhang et al. [49] obtained the reference genome of tartary buckwheat with high quality at the chromosome level, laying the foundation for our study that comprehensively analyzed *bZIP* family genes at the genome-wide level. In total, 96 *bZIP* genes were identified in tartary buckwheat (489 Mb) [49], and 75 *bZIPs* in *A. thaliana* (117 Mb) [10, 54], 63 in *Sesamum indicum* (sesame) (258 Mb) [55, 56], 89 in rice (466 Mb) [4, 57], 125 in maize (2182 Mb) [9, 58], 69 in tomato (900 Mb) [59, 60], and 247 in *Brassica napus* (rape) (844 Mb) [61, 62] were described in previous research. The number of *FtbZIP* genes is greater than the numbers of *bZIP* genes in *A. thaliana*, sesame, rice and tomato and fewer than those of maize and rape. There is no positive correlation between the number of *bZIP* genes and the genome size of these species. For example, among these species above, the number of *bZIP* genes in sesame is the least, but the genome size of sesame is not the smallest; the number in rape is the most, but the genome size is not the largest.

Phylogenetic analysis showed that the tartary buckwheat *bZIP* genes formed 11 groups by constructing an unrooted ML tree that analyzed and compared tartary buckwheat *bZIP* family members with *bZIPs* in *A. thaliana* (Fig. 1). In contrast with the number of groups in other angiosperms, the *FtbZIP* family has an identical number of groups (11) as *Salvia miltiorrhiza* [63] but more groups than cucumber and sorghum, which have 6 and 7 groups, respectively [17]. As described in the results, group H was not present in *A. thaliana*, so these two *FtbZIP* genes could not be classified. Thus we put these two *FtbZIP* genes alone in a group named H (Fig. 1).

By analyzing the gene structure and motif composition of the *FtbZIP* gene family (Fig. 2b and c), we can see that the 96 *FtbZIP* genes have some differences in gene structure; however, the members located in the same subfamily have similar gene structure. Groups E/H/K of the *FtbZIP* genes had no more than two introns, which provides a basis to prove the presumption that there may be a relationship between the low number of introns and stress response [4, 13, 64–66]. In addition, in terms of the results, although the motif constitutions have some differences among different groups, the motifs of the coded bZIP domain are quite conserved [62], and most bZIP proteins in the same subgroup have same motif constitutions. For example, all FtbZIP proteins in group F shares motifs 1, 2 and 5. Group B shares motifs 1, 3, 4, 5, 6 and 7 except FtPinG0008656300.01 that only contains motif 1. And group G possesses motifs 1, 2, 5 and 9 except FtPinG0005198100.01 only containing motif 5 and FtPinG0001576500.01 only lacking motif 5. Some specific motifs (such as motifs 4/8/9/10) only existing in specific groups may have various functions in the *bZIP* family of tartary buckwheat, which is coincident with the description of *bZIP* genes in sesame [56]. In summary, the phylogenetic analysis of the *FtbZIP* gene is consistent with gene structure; there is a similar structure in every subfamily, which was also reported for grape, *Malus domestica* (apple) and rape [62, 66, 67].

Tandem and segmental duplication play an important role in enriching protein function and promoting the evolution and expansion of gene families. Gene family expansion in plants through tandem or segmental duplication is important in the evolution of functional diversity [50]. In general, the tandem replication of genes forms a cluster of genes [68]. By analyzing the duplications of *FtbZIP* genes on 8 chromosomes (Figs. 3 and 4), we hypothesize that the emergence of some *FtbZIP* genes was probably caused by gene replication, and the evolution of *FtbZIP* genes is largely driven by these gene replication events. Comparing the intensity of the two duplication events, we conclude that segmental duplication is involved and important in expanding the *FtbZIP* genes, which is similar to the reports on rice and grape [4, 69]. This result also further revealed why tartary buckwheat has a high number of *bZIP* genes, similar to the study of *Brachypodium distachyon bZIP* genes [8].

The *bZIP* gene family plays a role in the growth and development crops, including developing flowers and maturing fruits [18, 21, 70]. We can hypothesize that most *FtbZIPs* may be related to growing and developing of tartary buckwheat by analyzing the expression patterns of *FtbZIP* family members in diverse plant tissues/organs, which is consistent with the discussion of former reported studies [13, 66, 71]. Despite the fact that a majority of the *FtbZIP* gene family members had broad expression in selected tissues/organs (Fig. 7), a number of *FtbZIP* genes exhibited distinct differences in expression among diverse organs, which was similarly demonstrated in previous reports about rice, maize and grape [4, 9, 69]. For example, *FtPinG00093625.01* is expressed only in tartary buckwheat root. The expression patterns of *FtPinG0003523300.01, FtPinG0008174200.01, FtPinG0009362500.01* and *FtPinG0001825500.01*, which are grouped into tartary buckwheat group A, are inconsistent, which means that each gene within a subfamily may to act differently. Similar results have also been described in grape [69]. In this article, based on *FtbZIP* genes homologous to *AtbZIP* genes according to the phylogenetic tree (Fig. 1), it is predicted that these *FtbZIP* genes may act consistent functions as their homologous *AtbZIP* genes. The functions of these *FtbZIP* genes need to be verified by experiments in the future, which lays a foundation for improving the growth and development process of tartary buckwheat crops. Plant pigments that sense light will induce nitrogen assimilation genes. Recently, it was found that the *bZIP TFs AtbZIP56/HY5* and *AtbZIP64/HYH* in *A. thaliana* play a positive role in the photoactivation of *nitrate reductase 2 (NIA2)*. Additionally, *NIA2* may be related to the cytosolic leaf localization of nitrate reductase [72]. *FtPinG0002935500.01,* which was homologous to *AtbZIP56/HY5* in the phylogenetic tree and was highly expressed in the tartary buckwheat leaf relatively, might be similar to *HY5* regarding the photoactivation of *NIA2*. In addition, *A. thaliana HY5* has been proven to be a basic leucine zipper transcription factor necessary for the photoregulation of cell extension and proliferation as well as chloroplast development [73, 74]. Photosynthesis in plants cannot be separated from the regulation of light. *FtPinG0002935500.01,* which was highly expressed in the leaf, may be related to leaf development. *FtPinG0009362500.01* was only expressed in the tartary buckwheat root, as shown in Fig. 7a. According to the motif composition analysis (Fig. 2c), *FtPinG0009362500.01* only has motif 5, unlike the other genes in group A. The specific tissue expression pattern of this gene may be related to its motif composition. Studies have also shown that the promoter of the *A. thaliana* gene contains motifs related to transcription factor binding sites specifically expressed in tissues and organs [75]. It has been reported that *AtbZIP1, AtbZIP2, AtbZIP44* and *AtbZIP53* from the *A. thaliana* group S are involved in balancing the supply and demand of carbohydrates [10]. Tartary buckwheat is a drought-tolerant species [76]. Regulating osmotic pressure through the accumulation of soluble sugars is the main physiological mechanism of drought tolerance in plants [77]. *FtPinG0006546100.01, FtPinG0005418500.01, FtPinG0002635300.01* and *FtPinG0006838500.01* from the tartary buckwheat group K, which are homologous to the members of the *A. thaliana* group S, have relatively high expression in the root (Fig. 7a). These *bZIP* genes probably have similar functions. This hypothesis can then be tested to prove whether these genes can be used to improve crop drought resistance and improve the species. Additionally, previous articles showed that the group S *bZIPs* are transcriptionally activated after stress (such as cold or drought) or expressed specifically in monocotyledonous and dicotyledonous flowers [78, 79]. The *FtbZIPs* from group K respectively show high expression in the root, stem, fruit and flower, suggesting that they may play wide regulatory roles in the development of tartary buckwheat like the *A. thaliana* homologues, which is similarly to a reported study in the rape [62].

Tartary buckwheat is a kind of plant with high nutritional and medicinal value. The total content of flavonoid compounds and proteins with balanced amino acids is greater than that of primary food crops [46]. The fruit is the main part of tartary buckwheat used medicinally. Additionally, *bZIP* genes regulate diverse biological processes, such as fruit development [80]. Thus, it is significant to research the expression of *FtbZIP* genes during fruit development (Fig. 8). The *AtbZIP TFs* in *A. thaliana* group C are thought to participate in important functional aspects such as fruit development [25]. *FtPinG0007900600.01* in tartary buckwheat group J, which was relatively highly expressed in the fruit, is homologous to the *bZIP* members of *A. thaliana* group C. Thus, these results will lay the foundation for us to further verify whether *FtPinG0007900600.01* has the same function as *A. thaliana* in fruit development.

Previous studies showed that ABA signaling plays major roles in the development and growth of plant tissues/organs, such as fruit, flower, root and seed [81, 82]. In recent years, there has also been increasing evidence that *bZIP* proteins are involved in phytohormone ABA signaling [83, 84]. According to a study on *A. thaliana,* the *bZIPs* from subgroup A, *AtbZIP39, AtbZIP36, AtbZIP38, AtbZIP35,* and *AtbZIP37*, play a major role in ABA signaling [10, 85, 86]. Thus, we can infer that the *FtbZIP* genes from group A are closely related to ABA signaling. As shown in Fig. 9a, ABA content has an increasing trend throughout the stages of tartary buckwheat fruit development (13 DAP to 25 DAP). Based on the results (Fig. 8), during fruit maturation, the expression of seven *FtbZIP* genes (*FtPinG0006838500.01*, *FtPinG0002753200.01*, *FtPinG0000068200.01, FtPinG0007266400.01*, *FtPinG0003523300.01*, *FtPinG0003196200.01* and *FtPinG0003227200.01*) was positively correlated with the variation of endogenous ABA content. Seven *FtbZIP* gene expression levels (*FtPinG0006546100.01, FtPinG0001825500.01, FtPinG0001861000.01, FtPinG0005418500.01, FtPinG0008174200.01, FtPinG0009407700.01* and *FtPinG0009423600.01)* were negatively correlated with the variation in endogenous ABA content. These *FtbZIP* genes may be closely related to the regulation of fruit development by ABA. *SlbZIP34/SlAREB1* has been thought to have regulatory roles in the primary metabolism pathways of tomato fruit [87]. This study concluded that the transcription factor *SlAREB1* might mediate ABA signaling to regulate fruit maturation by inducing the ethylene biosynthesis gene and changing cell wall metabolism. *FtPinG0003196200.01* homologous to *Solyc04g078840.2.1/ SlAREB1* (Fig. 6) has an increasing expression in tartary buckwheat fruit development stages, which is similar to *SlAREB1* [60]. The expression patterns of genes associated with ethylene production and cell wall modification in fruit development stages are related to *SlAREB1* expression. Thus, we can infer that *FtPinG0003196200.01* may also be involved in ABA signaling by regulating fruit ripening-related genes. *ABF2*, which is closely related to *FtPinG0003196200.01* evolution, is thought to perform important functions in seed germination and the sugar signaling pathway [88]. This makes our hypothesis more convincing. These results provide a basis for future experiments to verify the similarity of functional characteristics of the *FtbZIP* and *AtbZIP* genes in the growth and development of tartary buckwheat fruit.

Starch is the main storage substance of tartary buckwheat [38]. There was a positive correlation between the size and starch content of fruit and endogenous ABA content (Fig. 9a); ABA content is positively correlated with the fruit weight [38]. The increase in fruit weight (Fig. 9b) suggests that the exogenous application of ABA may affect starch accumulation and then the tartary buckwheat fruit weight. During embryonic development, ABA affects the biosynthesis of starch [89]. This is in line with our conjecture. The genes involved in ABA signal transduction regulate seed maturation and development [90]. ABA signal transduction requires a class of TF, bZIP, to perceive the ABA signal and then to regulate the expression of downstream genes by directly binding to the response element (ABRE) on the promoters of ABA-associated downstream genes, thus regulating the development of the embryo and endosperm during seed development [91, 92]. Members of the ABI5/ABF/AREB/DPBF subfamily form heteropolymers, which may regulate the same target genes and are functional redundant [93]. Therefore, we analyzed *FtPinG0003523300.01* and *FtPinG0003196200.01*, which were most closely related to *ABI5/ABF/AREB/DPBF* evolution. The expression levels of *FtPinG0003523300.01* and *FtPinG0003196200.01* during tartary buckwheat fruit development are positively correlated with the endogenous ABA content of fruit (13 DAP to 25 DAP). After the exogenous application of ABA, the expressions of these two genes in the fruit at 25 DAP decreased, which was negatively correlated with the increase of fruit weight. Therefore, ABA may regulate downstream gene expression by regulating the expression of *FtPinG0003523300.01* and *FtPinG0003196200.01*, thus indirectly affecting the fruit development of tartary buckwheat. These results provide an opportunity for us to further study the downstream gene regulation pathway of ABA signal transduction related to tartary buckwheat fruit development and then improve crop.

## Conclusion

We first identified and analyzed the genome-wide *FtbZIP TF* family in tartary buckwheat. We have identified 96 *FtbZIP* genes and analyzed their physical properties, evolutionary relationships, gene structures, conserved motifs, gene replication, expression patterns and changes following ABA treatment. Based on the above analyses and speculation of the functional characteristics of the *FtbZIP* family, we conclude that the *FtbZIP* genes have significant effects on tartary buckwheat development. We have identified two ABA-responsive genes, *FtPinG0003523300.01* and *FtPinG0003196200.01*, which are closely related to fruit development and maturity in tartary buckwheat, providing a theoretical basis for us to further explore the functional characteristics of tartary buckwheat through experiments and improve the crop yield of tartary buckwheat.

## Methods

### Identification of the *bZIP* family genes in tartary buckwheat

We downloaded the tartary buckwheat genome from the Tartary Buckwheat Genome Project (TBGP; http://www.mbkbase.org/Pinku1/). Tartary buckwheat *bZIP* sequences were obtained through two BLASTP methods. We chose the candidate sequences using the TBGP website and BLASTP search. We obtained the Hidden Markov Model (HMM) profile of the bZIP region with the Pfam protein family database (http://xfam.org/). *bZIPs* were identified using HMMER3.0 [94]. The existence of bZIP core sequences in candidate genes was confirmed by using the PFAM and SMART programs. In addition, we determined the CDS, MW and other properties of the identified bZIP proteins with the ExPasy website (https://web.expasy.org/compute_pi/).

### Phylogenetic analyses and classification of *FtbZIP* family members

The phylogenetic trees comparing tartary buckwheat and *A. thaliana* were constructed with the ML method. Multiple amino acid sequences of identified *bZIP* genes were aligned using MUSCLE [95]. In MEGA 7 [96], multiple ML trees were generated and the best-scoring tree was identified. We used the JTT + G + F model selected based on a ML model test. The ML phylogenetic tree was constructed with 1000 bootstrap replicates and assigned with bootstrap support values. All identified *FtbZIP* members were grouped into diverse subgroups on the basis of the divided subgroups of *AtbZIP*. The phylogenetic trees comparing tartary buckwheat and multiple species (*A. thaliana*, beet, tomato, grape and sunflower) were also constructed with the ML method above. The bZIP protein sequences (*A. thaliana*, beet, tomato, grape and sunflower) for ML phylogenetic trees were downloaded from the UniProt database (Available online: https://www.uniprot.org).

### intron structures/intron structures and conserved motifs in *FtbZIP* genes

To analyze the differences among the *FtbZIP* gene structures, we studied the conserved motifs in the encoded bZIP proteins. We analyzed the exon and intron constituents of the *FtbZIP* members with the Gene Structure Display Server (GSDS: http://gsds.cbi.pku.edu.cn) online tool. We used an online tool (http://meme.nbcr.net/meme/intro.html) to determine and analyze the proteins sequences of the *FtbZIP* family members with some parameters. We set the motif breadth to 6 ~ 200 and the number of motifs to 10 [48].

### Chromosomal spread and gene duplication of *FtbZIP* family members and evolutionary analysis with other species

We observed that all *FtbZIPs* genes were situated on tartary buckwheat LGs based on the information describing their location obtained from Circos [97]. We made use of Multiple collinear scanning toolkits (MCScanX) to detect the gene duplication events [98]. bZIP protein sequences in *A. thaliana*, beet, rice, sunflower, tomato, grape and soybean were obtained with the UniProt database (https://www.UniProt.org). We performed syntenic analyses on the homology of the *bZIP* family members in tartary buckwheat and other species with the Dual Systeny Plotter software [99].

### Plant materials

We collected the flower, stem, root, leaf and fruit of mature tartary buckwheat (XIQIAO) from the tartary buckwheat experimental base located at the farm of the Sichuan Agricultural University at three developmental stages, 13 (green fruit stage), 19 (expansion stage) and 25 (discoloration stage) DAP [38]. We kept the collected tissues/organs at − 80 °C for subsequent experiments.

### Exogenous ABA treatment and fruit weight determination

When tartary buckwheat was in the bud stage, plants with the same level of growth were treated with 2, 4, or 6 mg L^− 1^ ABA, while the control group was sprayed with the same amount of water. As the plants matured, we respectively determined the weights of 5 fruits at 13/19/25 DAP and confirmed the optimum concentration of ABA treatment. At 13 DAP, 19 DAP and 25 DAP, the fruits were individually picked and stored the samples at − 80 °C for subsequent experiments.

### Expression analyses of *FtbZIP* genes by qRT-PCR

The expression patterns of the *FtbZIP* genes identified in the different tissues (stem, root, leaf, flower, and fruit) and fruits at 25 DAP under ABA treatment were analyzed using qRT-PCR at least three times. The qRT-PCR primers of the *FtbZIP* genes listed in Additional file 4: Table S4 were obtained by the Primer3 software (http://frodo.wi.mit.edu/). We used the tartary buckwheat *H3* gene as the internal reference. The cDNA was produced with 1 mg RNA samples using a PrimeScript RT Reagent Kit with gDNA Eraser (TaKaRa) and SYBR Premix Ex Taq II (TaKaRa). The correlative expression data was calculated according to the 2^−(∆∆CT)^ method [100].

### Measurement of endogenous ABA content in fruit without ABA treatment

We ground fresh samples collected in liquid nitrogen. The powder was subsequently homogenized twice in methanol, evaporated, dissolved in water, decolorized, partitioned, evaporated and dissolved. We obtained the extract of the samples at different fruit development stages. The level of endogenous ABA in the samples was determined using high-performance liquid chromatography (HPLC) [38].

### Statistical analysis

We processed and analyzed all the above data with variance analysis using the Origin Pro 2018b (OriginLab Corporation, Northampton, Massachusetts, USA) statistics program and compared them with the least significant difference (LSD). We also used the Origin Pro 2018b to analyze the Pearson’s correlation coefficient, both for the expressions of *FtbZIP* genes, but also the expressions of *FtbZIP* genes and DAP.

## Additional files

Additional file 1: **Table S1.** List of the 96 *FtbZIP* genes identified in this study. (XLS 192 kb)

Additional file 2: **Table S2.** Analysis and distribution of the conserved motifs in tartary buckwheat bZIP proteins and other plants bZIP proteins. (XLS 36 kb)

Additional file 3: **Table S3.** One-to-one orthologous relationships between tartary buckwheat and other plants. (XLS 73 kb)

Additional file 4: **Table S4.** The primer sequences used for qRT-PCR. (XLS 32 kb)

## Data Availability

The genome sequences of tartary buckwheat used for identifying the *bZIP* genes in this study were located in the Tartary Buckwheat Genome Project (TBGP; http://www.mbkbase.org/Pinku1/). The tartary buckwheat accession (XIQIAO) materials used in the experiment were supplied by Professor Wang Anhu of Xichang University. All the datasets supporting the conclusions of this study are included in this article and its Additional files.

## Abbreviations

Aa: Amino acid
ABA: Abscisic acid
ABRE: ABA responsive element
*AtbZIP*: *A. thaliana bZIP*
bZIP: basic leucine zipper
CDS: Coding sequence length
DAP: Days after pollination
*FtbZIP* genes: *FtbZIPs*
*FtbZIP*: *Fagopyum talaricum bZIP*
GSDS: Gene Structure Display Server
HMM: Hidden Markov Model
HPLC: High-performance liquid chromatography
LGs: Linkage groups
LSD: Least significant difference
MCScan: Multiple collinear scanning
ML: Maximum likelihood
MW: Molecular weight
NIA2: Nitrate reductase 2
Pfam: Protein family
PI: Isoelectric point;
qRT-PCR: real-time Quantitative Polymerase Chain Reaction
TBGP: Tartary Buckwheat Genome Project
TF: Transcription factor

## References

[1] Riechmann JL, Ratcliffe OJ (2000). A genomic perspective on plant transcription factors. Curr Opin Plant Biol.

[2] Warren AJ (2002). Eukaryotic transcription factors. Curr Opin Struct Biol.

[3] Jinpu J, He Z, Lei K, Ge G, Jingchu L (2014). PlantTFDB 3.0: a portal for the functional and evolutionary study of plant transcription factors. Nucleic Acids Res.

[4] Aashima N, Mukesh J, Tyagi AK, Khurana JP (2008). Genomic survey and gene expression analysis of the basic leucine zipper transcription factor family in rice. Plant Physiol.

[5] Landschulz WH, Johnson PF, Mcknight SL (1988). The leucine zipper: a hypothetical structure common to a new class of DNA binding proteins. Science.

[6] Glover JN, Harrison SC (1995). Crystal structure of the heterodimeric bZIP transcription factor c-Fos-c-Jun bound to DNA. Nature.

[7] Wei H, Yang H, Yan Y, Wei Y, Tie W, Ding Z, Jiao Z, Ming P, Li K (2016). Genome-wide characterization and analysis of bZIP transcription factor gene family related to abiotic stress in cassava. Sci Rep.

[8] Xiang L, Chu Z (2015). Genome-wide evolutionary characterization and analysis of bZIP transcription factors and their expression profiles in response to multiple abiotic stresses in Brachypodium distachyon. BMC Genomics.

[9] Kaifa W, Juan C, Yanmei W, Yanhui C, Shaoxiang C, Yina L, Si P, Xiaojun Z, Daoxin X (2012). Genome-wide analysis of bZIP-encoding genes in maize. Dna Res An Int J Rapid Publ Rep Genes Genomes.

[10] Jakoby M, Weisshaar B, Dröge-Laser W, Vicente-Carbajosa J, Tiedemann J, Kroj T, Parcy F (2002). bZIP transcription factors in Arabidopsis. Trends Plant Sci.

[11] Riechmann JL, Heard J, Martin G, Reuber L, Jiang C, Keddie J, Adam L, Pineda O, Ratcliffe OJ, Samaha RR (2000). Arabidopsis transcription factors: genome-wide comparative analysis among eukaryotes. Science.

[12] Yáñez M, Cáceres S, Orellana S, Bastías A, Verdugo I, Ruiz-Lara S, Casaretto JA (2009). An abiotic stress-responsive bZIP transcription factor from wild and cultivated tomatoes regulates stress-related genes. Plant Cell Rep.

[13] Wang J, Zhou J, Zhang B, Vanitha J, Ramachandran S, Jiang SY (2011). Genome-wide expansion and expression divergence of the basic leucine zipper transcription factors in higher plants with an emphasis on Sorghum. J Integr Plant Biol.

[14] Wang XL, Chen X, Yang TB, Cheng Q, Cheng ZM (2017). Genome-Wide Identification of bZIP Family Genes Involved in Drought and Heat Stresses in Strawberry (*Fragaria vesca*). Int J Genomics.

[15] Que F, Wang GL, Huang Y, Xu ZS, Wang F, Xiong AS (2015). Genomic identification of group a bZIP transcription factors and their responses to abiotic stress in carrot. Genet Mol Res.

[16] Pourabed E, Golmohamadi FG, Monfared PS, Razavi SM, Shobbar ZS (2015). Basic leucine zipper family in barley: genome-wide characterization of members and expression analysis. Mol Biotechnol.

[17] Mehmet Cengiz Baloglu VE, Hajyzadeh M, Unver T (2014). Genome-wide analysis of the bZIP transcription factors in cucumber. PLoS One.

[18] Zhengwei J, Wei X, Aizhong L (2014). Genomic surveys and expression analysis of bZIP gene family in castor bean (Ricinus communis L.). Planta.

[19] Fukazawa J, Sakai T, Ishida S, Yamaguchi I, Kamiya Y, Takahashi Y (2000). Repression of shoot Growth, a bZIP transcriptional activator, regulatescell elongation by controlling the level of gibberellins. Plant Cell.

[20] Yin Y, Zhu Q, Dai S, Lamb C, Beachy RN (1997). RF2a, a bZIP transcriptional activator of the phloem-specific rice tungro bacilliform virus promoter, functions in vascular development. EMBO J.

[21] Abe M, Kobayashi Y, Yamamoto S, Daimon Y, Yamaguchi A, Ikeda Y, Ichinoki H, Notaguchi M, Goto K, Araki T (2005). FD, a bZIP protein mediating signals from the floral pathway integrator FT at the shoot apex. Science.

[22] Silveira AB, Gauer L, Tomaz JP, Cardoso PR, Carmello-Guerreiro S, Vincentz M (2007). The Arabidopsis AtbZIP9 protein fused to the VP16 transcriptional activation domain alters leaf and vascular development. Plant Sci.

[23] Huaishun S, Kaiming C, Xiping W (2007). A conserved proline residue in the leucine zipper region of AtbZIP34 and AtbZIP61 in Arabidopsis thaliana interferes with the formation of homodimer. Biochem Biophys Res Commun.

[24] Elena BG, Filip R, Thevelein JM, Jen S (2007). A central integrator of transcription networks in plant stress and energy signalling. Nature.

[25] Pilar L, Luis O-S, Zamira A, Cristina F, Isabel D, Pilar C, Jesúus V-C (2003). Synergistic activation of seed storage protein gene expression in Arabidopsis by ABI3 and two bZIPs related to OPAQUE2. J Biol Chem.

[26] Claudia N, Busk PK, Eva DP, Victoria L, Testillano PS, Maria-Carmen RO, Montserrat P (2005). Isolation and functional characterisation of two new bZIP maize regulators of the ABA responsive gene rab28. Plant Mol Biol.

[27] Uno Y, Furihata T, Abe H, Yoshida R, Shinozaki K, Yamaguchi-Shinozaki K (2000). Arabidopsis basic leucine zipper transcription factors involved in an abscisic acid-dependent signal transduction pathway under drought and high-salinity conditions. Proc Natl Acad Sci U S A.

[28] Wellmer F, Kircher S, Rügner A, Frohnmeyer H, Schäfer E, Harter K (1999). Phosphorylation of the parsley bZIP transcription factor CPRF2 is regulated by light. J Biol Chem.

[29] Roman U, Alexander B, Attila O, Zoltán M, Eva A, Oakeley EJ, Eberhard SF, Ferenc N (2004). Genome-wide analysis of gene expression reveals function of the bZIP transcription factor HY5 in the UV-B response of Arabidopsis. Proc Natl Acad Sci U S A.

[30] Ying S, Zhang DF, Fu J, Shi YS, Song YC, Wang TY (2012). Cloning and characterization of a maize bZIP transcription factor, ZmbZIP72, confers drought and salt tolerance in transgenic Arabidopsis. Planta.

[31] Liu C, Mao B, Ou S, Wang W, Liu L, Wu Y, Chu C, Wang X (2014). OsbZIP71, a bZIP transcription factor, confers salinity and drought tolerance in rice. Plant Mol Biol.

[32] Corinna T, Andreas S, Stefanie K, Thomas B, Kaloian N, Christiane G (2010). Tobacco bZIP transcription factor TGA2.2 and related factor TGA2.1 have distinct roles in plant defense responses and plant development. Plant J.

[33] Hironori K, Christian NK, Petra E, Jan D, Katia S, Christina C, Holt BF, Thomas M, Eberhard SF, Klaus H (2014). bZIP10-LSD1 antagonism modulates basal defense and cell death in Arabidopsis following infection. EMBO J.

[34] Finkelstein RR, Lynch TJ (2000). The Arabidopsis abscisic acid response gene ABI5 encodes a basic leucine zipper transcription factor. Plant Cell.

[35] Choi H, Hong J, Ha J, Kang J, Kim SY (2000). ABFs, A family of ABA-responsive element binding factors. J Biol Chem.

[36] Sandra B, Sonia R, Guillaume L, Delphine J, Véronique P, Fabienne G, Jérôme G, François P (2002). The homologous ABI5 and EEL transcription factors function antagonistically to fine-tune gene expression during late embryogenesis. Plant Cell.

[37] Jose C, Tuan-Hua David H (2003). The transcription factors HvABI5 and HvVP1 are required for the abscisic acid induction of gene expression in barley aleurone cells. Plant Cell.

[38] Liu M, Ma Z, Zheng T, Sun W, Zhang Y, Jin W, Zhan J, Cai Y, Tang Y, Wu Q (2018). Insights into the correlation between physiological changes in and seed development of tartary buckwheat (*Fagopyrum tataricum* Gaertn). BMC Genomics.

[39] Hu YF, Li YP, Zhang J, Liu H, Chen Z, Huang Y (2011). PzsS3a ,a novel endosperm specific promoter from maize ( *Zea mays* L.) induced by ABA. Biotechnol Lett.

[40] Holasova M, Fiedlerova V, Smrcinova H, Orsak M, Lachman J, Vavreinova S (2002). Buckwheat-the source of antioxidant activity in functional foods. Food Res Int.

[41] Duarte R, Carvalho A, Gadelha D, Braga V (2014). Rutin reduces oxidative stress in animals with renovascular hypertension. BMC Proc.

[42] Wójcicki J, Barcew-Wiszniewska B, Samochowiec L, Rózewicka L (1995). Extractum Fagopyri reduces atherosclerosis in high-fat diet fed rabbits. Pharmazie.

[43] Watanabe M (1998). Catechins as antioxidants from buckwheat (Fagopyrum esculentum Moench) groats. J Agric Food Chem.

[44] Ohsawa R, Tsutsumi T (1995). Inter-varietal variations of rutin content in common buckwheat flour (Fagopyrum esculentum Moench). Euphytica.

[45] Bonafaccia G, Marocchini M, Kreft I (2003). Composition and technological properties of the flour and bran from common and tartary buckwheat. Food Chem.

[46] Nina F, Janko R, Iztok Joze K, Zhuanhua W, Zheng Z, Ivan K (2003). Tartary buckwheat (Fagopyrum tataricum Gaertn.) as a source of dietary rutin and quercitrin. J Agric Food Chem.

[47] Liu M, Fu Q, Ma Z, Sun W, Huang L, Wu Q, Tang Z, Bu T, Li C, Chen H (2019). Genome-wide investigation of the MADS gene family and dehulling genes in tartary buckwheat (Fagopyrum tataricum). Planta.

[48] Liu M, Ma Z, Wang A, Zheng T, Huang L, Sun W, Zhang Y, Jin W, Zhan J, Cai Y (2018). Genome-wide investigation of the auxin response factor gene family in tartary buckwheat (Fagopyrum tataricum). Int J Mol Sci.

[49] Zhang L, Li X, Ma B, Gao Q, Du H, Han Y, Li Y, Cao Y, Qi M, Zhu Y (2017). The Tartary buckwheat genome provides insights into Rutin biosynthesis and abiotic stress tolerance. Mol Plant.

[50] Cannon SB, Mitra A, Baumgarten A, Young ND, May G (2004). The roles of segmental and tandem gene duplication in the evolution of large gene families in Arabidopsis thaliana. BMC Plant Biol.

[51] Katia S (2008). Post-translational regulation of plant bZIP factors. Trends Plant Sci.

[52] Liu M, Ma Z, Zheng T, Wang J, Huang L, Sun W, Zhang Y, Jin W, Zhan J, Cai Y (2018). The potential role of auxin and abscisic acid balance and FtARF2 in the final size determination of tartary buckwheat fruit. Int J Mol Sci.

[53] Song C, Xiang DB, Yan L, Song Y, Zhao G, Wang YH, Zhang BL (2016). Changes in seed growth, levels and distribution of flavonoids during tartary buckwheat seed development. Plant Prod Sci.

[54] Zapata L, Jia D, Willing EM, Hartwig B, Bezdan D, Jiao WB, Patel V, James GV, Koornneef M, Ossowski S (2016). Chromosome-level assembly of arabidopsis thaliana ler reveals the extent of translocation and inversion polymorphisms. Proc Natl Acad Sci U S A.

[55] Wang L, Xia Q, Zhang Y, Zhu X, Zhu X, Li D, Ni X, Gao Y, Xiang H, Wei X (2016). Updated sesame genome assembly and fine mapping of plant height and seed coat color QTLs using a new high-density genetic map. BMC Genomics.

[56] Wang Y, Zhang Y, Zhou R, Dossa K, Yu J, Li D, Liu A, Mmadi MA, Zhang X, You J (2018). Identification and characterization of the bZIP transcription factor family and its expression in response to abiotic stresses in sesame. PLoS One.

[57] Yu J, Hu S, Wang J, Li S, Wong KSG, Liu B, Deng Y, Li D, Yan Z, Zhang X (2001). A draft sequence of the rice (Oryza sativa ssp. indica) genome. Chin Sci Bull.

[58] Sun S, Zhou Y, Chen J, Shi J, Zhao H, Zhao H, Song W, Zhang M, Cui Y, Dong X (2018). Extensive intraspecific gene order and gene structural variations between Mo17 and other maize genomes. Nat Genet.

[59] Sato S, Tabata S, Hirakawa H, Asamizu E, Shirasawa K, Isobe S, Kaneko T, Nakamura Y, Shibata D, Aoki K (2012). The tomato genome sequence provides insights into fleshy fruit evolution. Nature.

[60] Li D, Fu F, Zhang H, Song F (2015). Genome-wide systematic characterization of the bZIP transcriptional factor family in tomato (Solanum lycopersicum L). BMC Genomics.

[61] Boulos C, France D, Shengyi L, Parkin IAP, Haibao T, Xiyin W, Julien C, Harry B, Chaobo T, Birgit S (2014). Plant genetics. Early allopolyploid evolution in the post-neolithic brassica napus oilseed genome. Science.

[62] Zhou Y, Xu D, Jia L, Huang X, Ma G, Wang S, Zhu M, Zhang A, Guan M, Lu K (2017). Genome-wide identification and structural analysis of bZIP transcription factor genes in brassica napus. Genes.

[63] Zhang Y, Xu Z, Ji A, Luo H, Song J, Zhang Y, Xu Z, Ji A, Luo H, Song J (2018). Genomic survey of bZIP transcription factor genes related to tanshinone biosynthesis inSalvia miltiorrhiza. Acta Pharm Sin B.

[64] Lan T, Gao J, Zeng QY (2013). Genome-wide analysis of the LEA (late embryogenesis abundant) protein gene family in Populus trichocarpa. Tree Genet Genomes.

[65] Yu L, Xiong Z, Zheng J, Xu D, Zhu Z, Xiang J, Gan J, Raboanatahiry N, Yin Y, Li M (2016). Genome-wide identification, structural analysis and new insights into late embryogenesis abundant (LEA) gene family formation pattern in Brassica napus. Sci Rep.

[66] Zhao J, Guo R, Guo C, Hou H, Wang X, Gao H (2016). Evolutionary and expression analyses of the apple basic leucine zipper transcription factor family. Front Plant Sci.

[67] Gao M, Zhang H, Guo C, Cheng C, Guo R, Mao L, Fei Z, Wang X (2014). Evolutionary and expression analyses of basic zipper transcription factors in the highly homozygous model grape PN40024 ( Vitis vinifera L.). Plant Mol Biol Report.

[68] Savard OT, Bertrand D, El-Mabrouk N (2011). Evolution of orthologous tandemly arrayed gene clusters. Bmc Bioinf.

[69] Liu J, Chen N, Chen F, Cai B, Santo SD, Tornielli GB, Pezzotti M, Cheng ZM (2014). Genome-wide analysis and expression profile of the bZIP transcription factor gene family in grapevine (Vitis vinifera ). BMC Genomics.

[70] Xu Z, Ali Z, Xu L, He X, Huang Y, Yi J, Shao H, Ma H, Zhang D (2016). The nuclear protein GmbZIP110 has transcription activation activity and plays important roles in the response to salinity stress in soybean. Sci Rep.

[71] Hou Y, Wu A, He Y, Li F, Wei C (2018). Genome-wide characterization of the basic leucine zipper transcription factors in camellia sinensis. Tree Genet Genomes.

[72] Jonassen EM, Sévin DC, Lillo C (2009). The bZIP transcription factors HY5 and HYH are positive regulators of the main nitrate reductase gene in Arabidopsis leaves, NIA2 , but negative regulators of the nitrate uptake gene NRT1.1. J Plant Physiol.

[73] Chattopadhyay S, Ang LH, Puente P, Deng XW, Wei N (1998). Arabidopsis bZIP protein HY5 directly interacts with light-responsive promoters in mediating light control of gene expression. Plant Cell.

[74] Tokitaka O, Yoshiro S, Kiyotaka O (1997). The Arabidopsis HY5 gene encodes a bZIP protein that regulates stimulus-induced development of root and hypocotyl. Genes Dev.

[75] Nitz I, Berkefeld H, Puzio PS, Grundler FMW (2001). Pyk10, a seedling and root specific gene and promoter from *Arabidopsis thaliana*. Plant Sci.

[76] Gao F, Zhou J, Deng RY, Zhao HX, Li CL, Chen H, Suzuki T, Park SU, Wu Q (2017). Overexpression of a tartary buckwheat R2R3-MYB transcription factor gene, FtMYB9, enhances tolerance to drought and salt stresses in transgenic arabidopsis. J Plant Physiol.

[77] Silva EN, Ferreira-Silva SL, Viegas RA, Gomes Silveira JA (2010). The role of organic and inorganic solutes in the osmotic adjustment of drought-stressed Jatropha curcas plants. Environ Exp Bot.

[78] Martínez-García JF, Moyano E, Alcocer MJ, Martin C (2010). Two bZIP proteins from Antirrhinum flowers preferentially bind a hybrid C-box/G-box motif and help to define a new sub-family of bZIP transcription factors. Plant J.

[79] Strathmann A, Kuhlmann M, Heinekamp T, Dröge-Laser W (2010). BZI-1 specifically heterodimerises with the tobacco bZIP transcription factors BZI-2, BZI-3/TBZF and BZI-4, and is functionally involved in flower development. Plant J.

[80] Masayuki PY, Yasuyuki O, Satoru MT, Fumio T (2006). Synergism between RPBF Dof and RISBZ1 bZIP activators in the regulation of rice seed expression genes. Plant Physiol.

[81] Wan-Hsing C, Akira E, Li Z, Jessica P, Huei-Chi C, Analilia A, Patricia L, Eiji N, Tadao A, Mitsunori S (2002). A unique short-chain dehydrogenase/reductase in Arabidopsis glucose signaling and abscisic acid biosynthesis and functions. Plant Cell.

[82] Ying Z, Adams IP, Colin R (2007). Malic enzyme: the controlling activity for lipid production? Overexpression of malic enzyme in mucor circinelloides leads to a 2.5-fold increase in lipid accumulation. Microbiol.

[83] Xiang Y, Tang N, Du H, Ye H, Xiong L (2008). Characterization of OsbZIP23 as a key player of the basic leucine zipper transcription factor family for conferring abscisic acid sensitivity and salinity and drought tolerance in rice. Plant Physiol.

[84] Lu G, Gao C, Zheng X, Han B (2009). Identification of OsbZIP72 as a positive regulator of ABA response and drought tolerance in rice. Planta.

[85] Hossain MA, Cho JI, Han M, Ahn CH, Jeon JS, An G, Park PB (2010). The ABRE-binding bZIP transcription factor OsABF2 is a positive regulator of abiotic stress and ABA signaling in rice. J Plant Physiol.

[86] Yucheng G, Haibo R, He X, Zeyang M, Fan C (2010). Identification and characterization of bZIP-type transcription factors involved in carrot (Daucus carota L.) somatic embryogenesis. Plant J.

[87] Bastías A, Yañez M, Osorio S, Arbona V, Gómezcadenas A, Fernie AR, Casaretto JA (2014). The transcription factor AREB1 regulates primary metabolic pathways in tomato fruits. J Exp Bot.

[88] Kim S, Kang JY, Cho DI, Park JH, Kim SY (2010). ABF2, an ABRE-binding bZIP factor, is an essential component of glucose signaling and its overexpression affects multiple stress tolerance. Plant J.

[89] Crouch ML, Sussex IM (1981). Development and storage-protein synthesis in Brassica napus L. embryos in vivo and in vitro. Planta.

[90] Finkelstein RR, Gampala SSL, Rock CD (2002). Abscisic Acid Signaling in Seeds and Seedlings. Plant Cell.

[91] Shao X. Molecular basis of abscisic acid (ABA) regulating early development of *Arabidopsis thaliana* seeds. Shandong: Shandong Agricultural University; 2015.

[92] Hubbard KE, Noriyuki N, Kenichi H, Getzoff ED, Schroeder JI (2010). Early abscisic acid signal transduction mechanisms: newly discovered components and newly emerging questions. Genes Dev.

[93] Kim SY, Chung HJ, Thomas TL (2010). Isolation of a novel class of bZIP transcription factors that interact with ABA-responsive and embryo-specification elements in the Dc3 promoter using a modified yeast one-hybrid system. Plant J.

[94] Eddy SR. Profile Hidden Markov Models. 1998;14:755–63. 10.1093/bioinformatics/14.9.755 9918945

[95] Edgar RC (2004). MUSCLE: multiple sequence alignment with high accuracy and high throughput. Nucleic Acids Res.

[96] Kumar S, Stecher G, Tamura K (2016). MEGA7: molecular evolutionary genetics analysis version 7.0 for bigger datasets. Mol Biol Evol.

[97] Krzywinski M, Schein JI (2009). Circos: an information aesthetic for comparative genomics. Genome Res.

[98] Yupeng W, Haibao T, Jeremy DD, Xu T, Jingping L, Xiyin W, Tae-ho L, Huizhe J, Barry M, Hui G. MCScanX: a toolkit for detection and evolutionary analysis of gene synteny and collinearity. 2012;40(7):e49. 10.1093/nar/gkr1293 PMC3326336 22217600

[99] Liu C, Xie T, Chen C, Luan A, Long J, Li C, Ding Y, He Y (2017). Genome-wide organization and expression profiling of the R2R3-MYB transcription factor family in pineapple ( Ananas comosus ). BMC Genomics.

[100] Livak KJ, Schmittgen TD (2001). Analysis of relative gene expression data using real-time quantitative PCR and the 2 −ΔΔ C T method. Methods.

